# Supplementary material for: TMEM97/Sigma 2 Receptor Increases Estrogen Receptor α Activity in Promoting Breast Cancer Cell Growth
Source: Cancers (Basel). 2023 Dec 2;15(23):5691. doi: 10.3390/cancers15235691 (PMC10705716; doi:10.3390/cancers15235691)
Supplement: Supplementary file 1 [file cancers-15-05691-s001.zip › cancers-2738446-supplementary.pdf]

TMEM97/Sigma 2 Receptor Increases Estrogen Receptor α Activity in Promoting Breast Cancer Cell Growth

Yuanqin Zhang, Xiangwei Fang, Jiuhui Wang and Daotai Nie

Supplemental Figure

(A) **(B)**

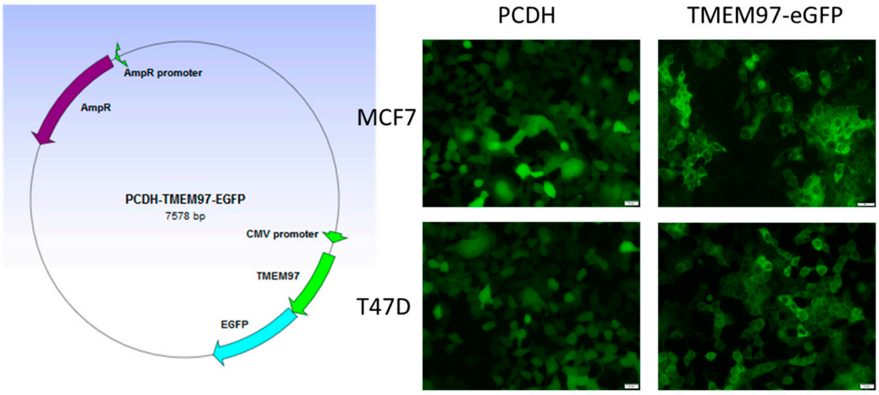

(C)

Clone ID:	Vector Map	Targets
V3LHS_333131	pGIPZ	ORF
V2LHS_54836	pGIPZ	3'UTR
V3LHS_404602	pGIPZ	3'UTR

Supplemental Figure S1

(**A**). The plasmid map of TMEM97-EGFP on PCDH-CMV vector to generate TMEM97 stable overexpression breast cancer cells.

(B). Visualization of TMEM97eGFP cells in MCF7 and T47D compared to vector control.

(C). Commercial modified miR-30 based GIPZ Lentiviral shRNA used for TMEM97 knockdown.

Figure 2

(A)

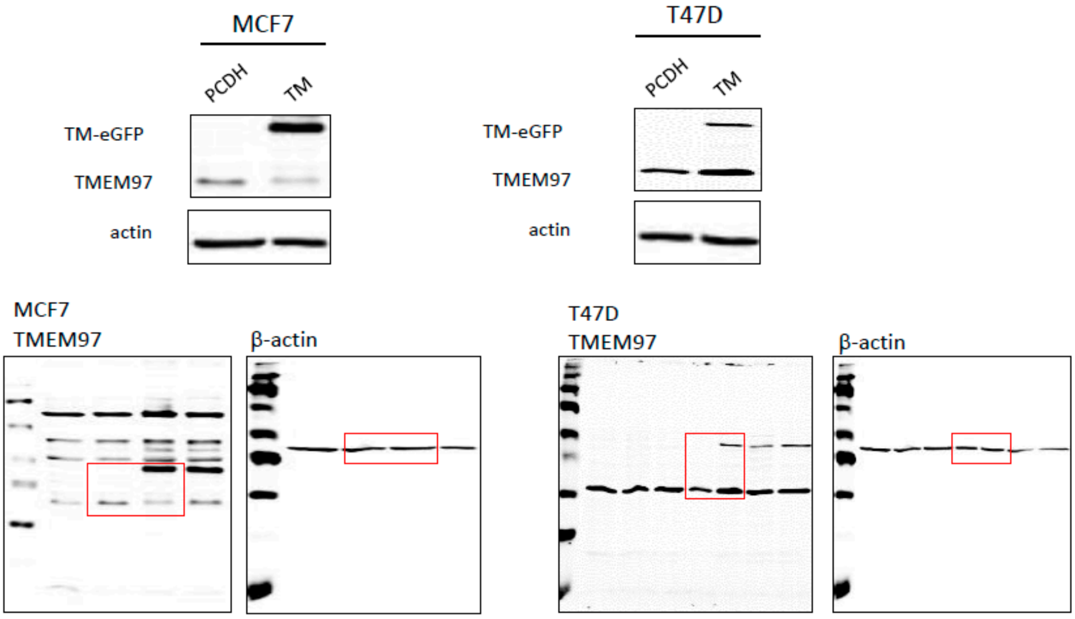

Original blots of Figure 2.

Figure 4

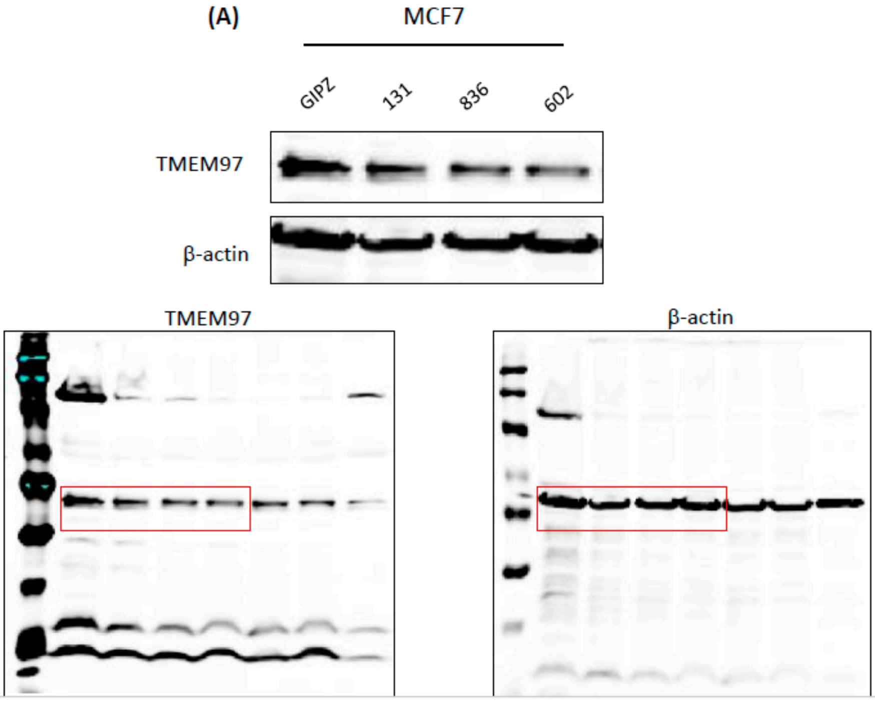

Original blots of Figure 4.

Figure 6

(A)

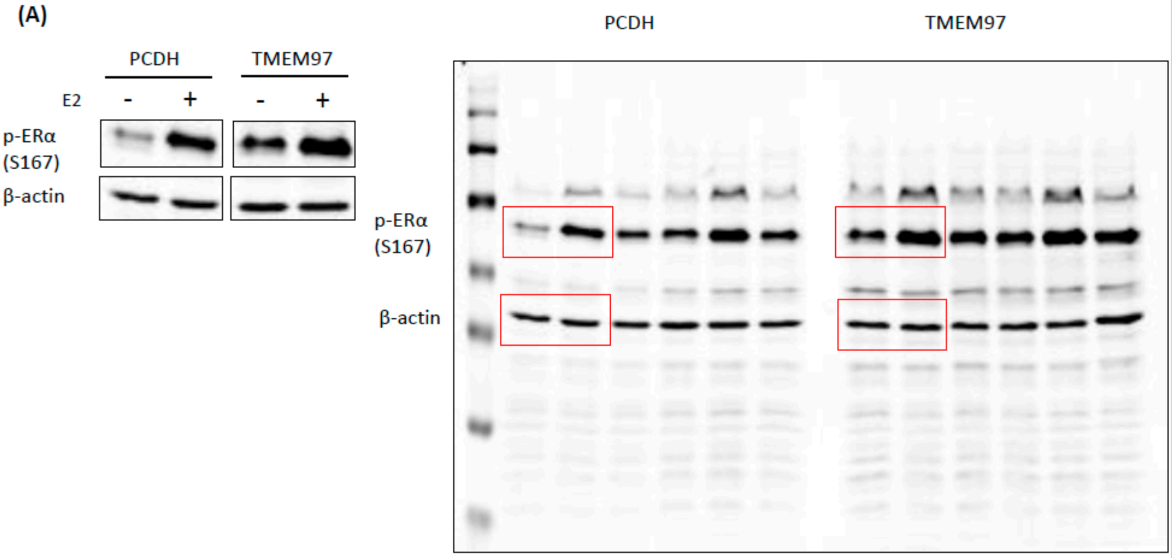

Figure 6

(C)

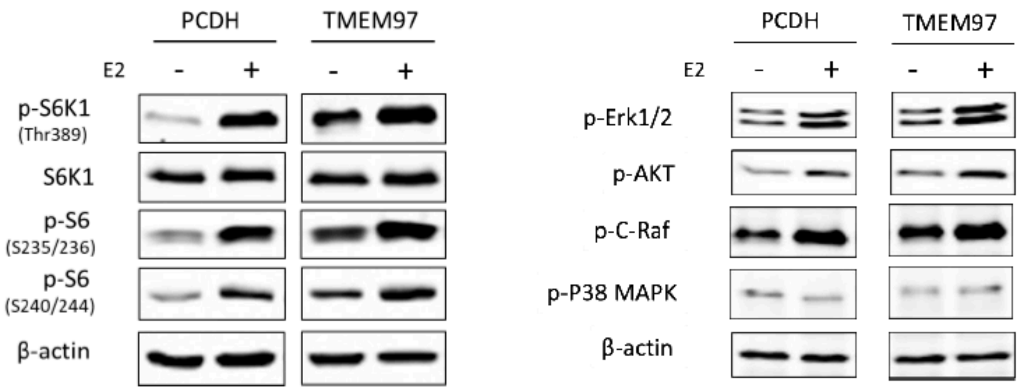

Figure 6

(C)

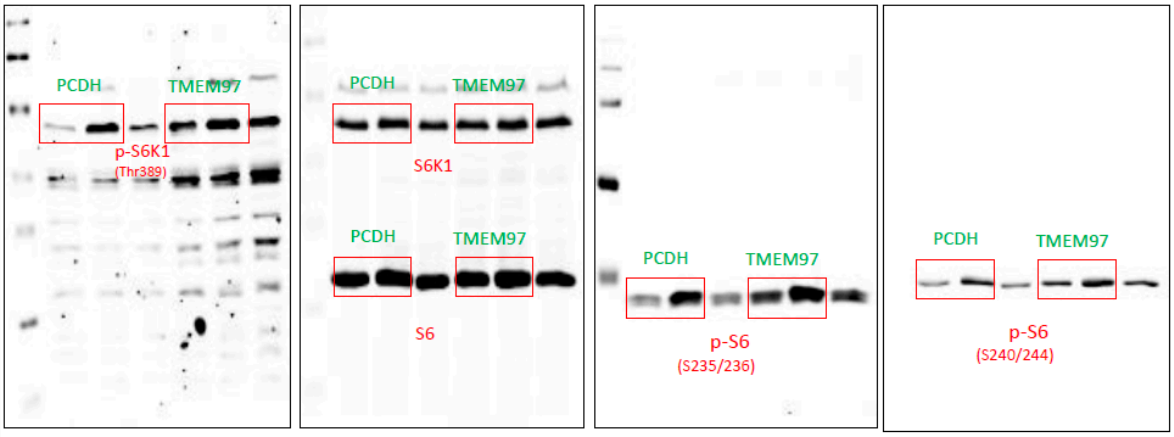

Figure 6

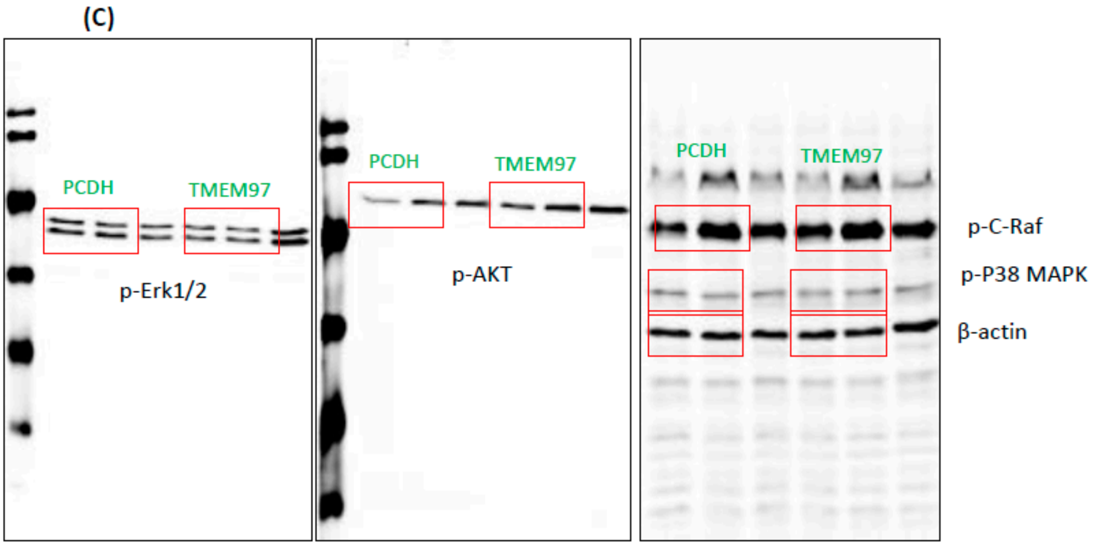

Figure 6

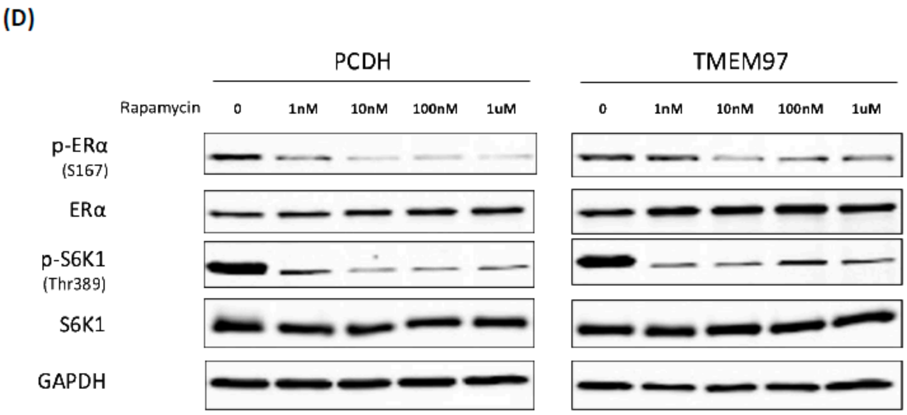

Figure 6

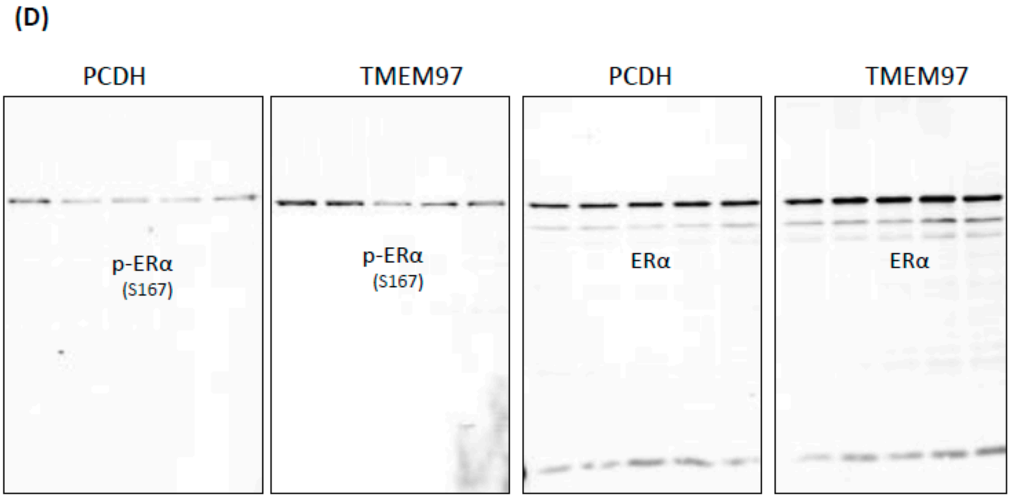

Figure 6 (D)

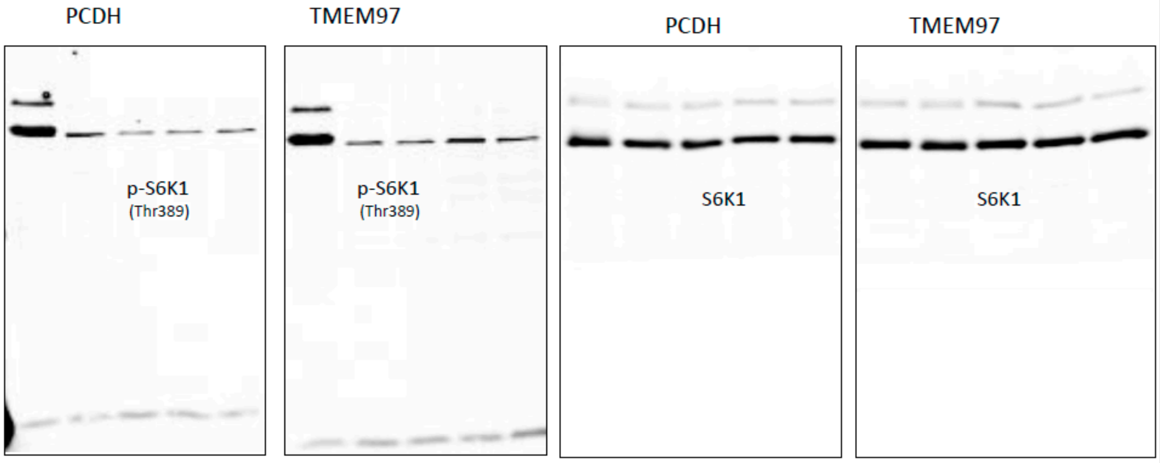

Figure 6 (D)

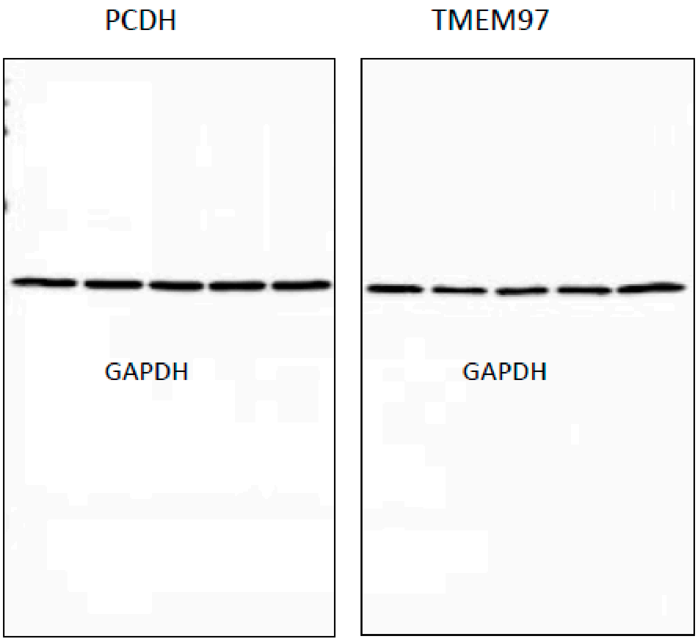

Original blots of Figure 6.

Figure 7

(B)

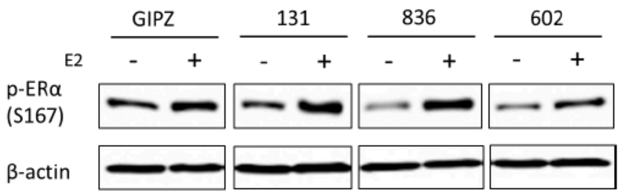

Figure 7 (B)

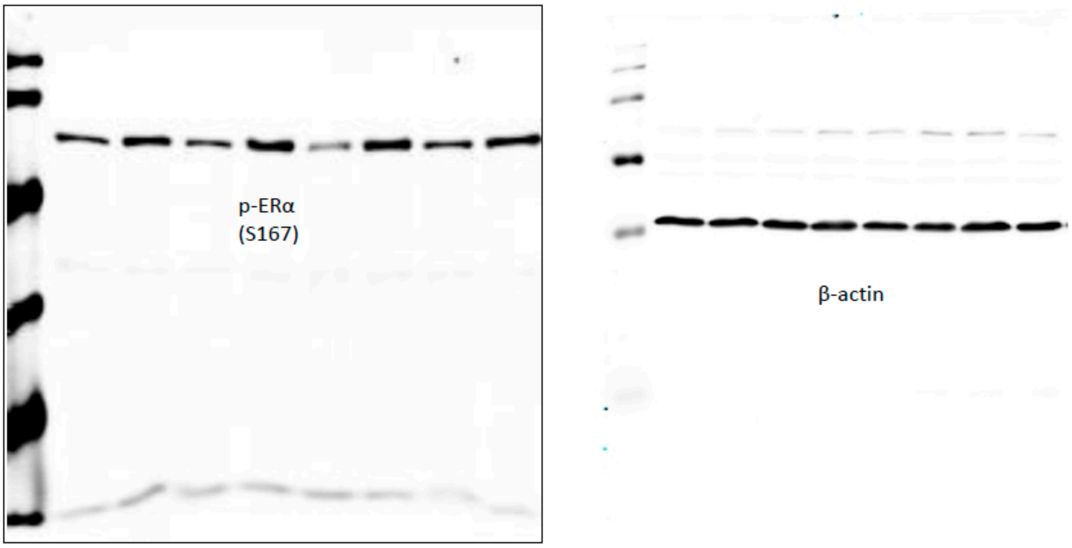

Figure 7

(C)

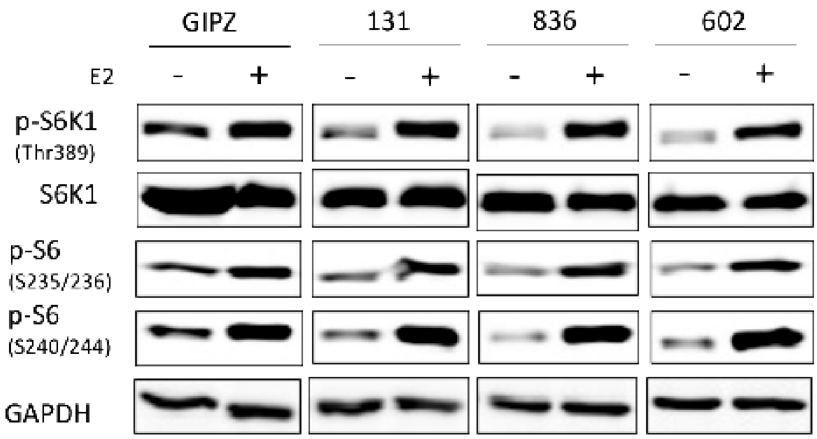

Figure 7 (C)

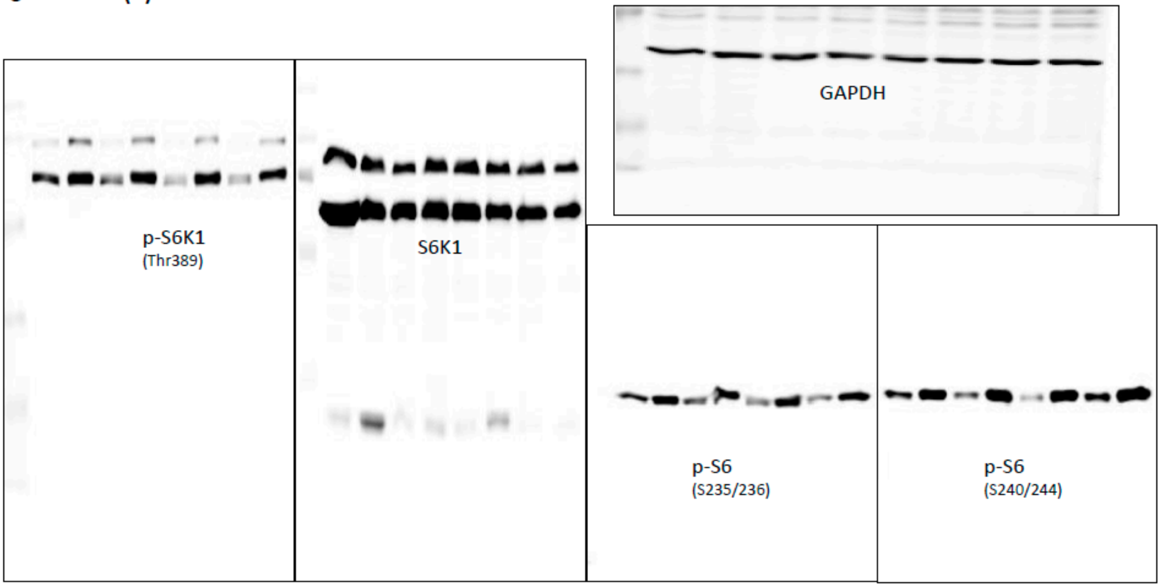

Original blots of Figure 7.
